# Early postoperative voice-change phenotypes after thyroid surgery: a prospective cohort study

**DOI:** 10.3389/fendo.2026.1845546

**Published:** 2026-06-15

**Authors:** Heyang Jiao, Yingying Wang, Shijie Li, Peiyao Wang, Jiedong Kou, Yuqing Gao, Yishen Zhao, Hui Sun

**Affiliations:** 1 Department of Thyroid Surgery, The China-Japan Union Hospital of Jilin University, Jilin Provincial Key Laboratory of Thyroid Disease, Changchun, China; 2 Jilin Provincial Precision Medicine Laboratory of Molecular Biology and Translational Medicine On Differentiated Thyroid Carcinoma, Changchun, China

**Keywords:** acoustic analysis, patient-reported voice handicap, perceptual voice assessment, postoperative voice change, spectral features, thyroid surgery, unsupervised clustering, voice phenotyping

## Abstract

**Background:**

Post-thyroidectomy voice change is common, but early postoperative objective voice-change patterns and their short-term clinical relevance remain insufficiently characterized.

**Methods:**

In this single-center prospective cohort study, patients undergoing thyroid surgery were screened consecutively from October 2025 to November 2025. Standardized voice recordings were obtained at baseline and postoperative day 2 (POD2), and clinical voice outcomes were assessed at postoperative day 7 (POD7). Five POD2 spectral change features relative to baseline were used to derive phenotypes through a prespecified two-stage unsupervised clustering workflow based on partitioning around medoids, Manhattan distance, and silhouette-based cluster-number selection. Clinical validation used change in Voice Handicap Index-30 (VHI-30), the prespecified VHI-30 responder definition (Delta VHI-30 ≥ 13), and change in Grade within the Grade, Roughness, Breathiness, Asthenia, Strain scale. Robustness was evaluated using consensus clustering, proportion of ambiguous clustering, sample-level stability, and resampling-based adjusted Rand index reproducibility. Sensitivity analyses compared the primary workflow with one-step k = 3 and k = 4 clustering alternatives, and exploratory analyses evaluated operative and demographic factors in relation to POD7 outcomes.

**Results:**

Of 401 screened patients, 245 were included in the final analytic cohort. The final phenotypes were A/B/C = 59/56/130. All five spectral clustering features differed significantly across phenotypes (all P < 0.001). At POD7, phenotypes differed significantly in VHI-30 change (epsilon-squared = 0.641), VHI-30 responder rate (Cramér’s V = 0.639), and Grade change (epsilon-squared = 0.203) (all P < 0.001). Phenotype B showed the greatest short-term burden, including a responder rate of 53.6% (30/56; exact 95% confidence interval, 39.7%–67.0%), compared with 1.7% (1/59; exact 95% confidence interval, 0.0%–9.1%) in phenotype A and 1.5% (2/130; exact 95% confidence interval, 0.2%–5.4%) in phenotype C. Sensitivity analyses did not support replacing the prespecified two-stage workflow with one-step k = 3 or k = 4 alternatives. Exploratory analyses suggested that the clinical profile of phenotype B was not simply explained by thyroidectomy extent, surgical approach, lateral neck dissection, or operative time.

**Conclusions:**

Early postoperative objective voice changes after thyroid surgery can be organized into clinically interpretable short-term phenotypes associated with patient-reported and supportive perceptual outcomes. These phenotypes should be interpreted as early postoperative voice-change patterns rather than as nerve-injury phenotypes or persistent long-term voice categories.

## Introduction

Post-thyroidectomy voice change is common and clinically important. Even in the absence of overt recurrent laryngeal nerve (RLN) injury, patients may experience new or worsened voice problems after thyroid surgery (1–4). Papadakis et al. reported significant early postoperative changes in multiple subjective and objective voice measures after total thyroidectomy (1). Tedla et al. similarly found short-term worsening in patient-reported voice symptoms and in perceptual Grade, Roughness, Breathiness, Asthenia, Strain (GRBAS) ratings after thyroidectomy without superior or recurrent laryngeal nerve injury (2). Hong et al. further demonstrated significant postoperative changes in speech-related acoustic parameters in patients without laryngeal nerve injury (3). Together, these findings indicate that post-thyroidectomy dysphonia cannot be understood solely as a consequence of frank RLN paralysis. Intubation-related irritation, tissue edema, strap-muscle dysfunction, altered laryngotracheal mechanics, and subtle neural or non-neural perioperative factors may all contribute (3, 4).

Postoperative voice changes also have a time-dependent course. Early postoperative deterioration may improve during recovery, whereas a subset of patients may continue to experience persistent or delayed voice-related symptoms (5). Therefore, assessments performed during the first postoperative week are useful for identifying early postoperative burden, but they cannot by themselves distinguish transient postoperative changes from persistent voice dysfunction. This is particularly relevant because early recordings may still be influenced by edema, intubation-related effects, pain, temporary neuromuscular changes, or protective voice use.

This complexity also means that no single measure is sufficient. Contemporary voice evaluation typically combines patient-reported outcomes, clinician-rated perceptual scales, and objective acoustic analysis. The Voice Handicap Index-30 (VHI-30) is one of the most widely used patient-reported tools for dysphonia and captures functional, physical, and emotional aspects of voice handicap (6). Perceptual evaluation using the GRBAS scale provides complementary information on overall dysphonia severity and voice quality (7). In the present study, patient-reported assessment used the Mandarin Chinese version of the VHI-30 (8). Prior work has shown that patient-reported handicap and laboratory-based voice measures are related but not redundant (9). Accordingly, multidimensional assessment is recommended when evaluating voice disorders and treatment-related voice outcomes (10).

Objective assessment has also moved beyond traditional perturbation measures alone. In addition to conventional acoustic variables, recent work has emphasized spectral and cepstral analyses as sensitive markers of postoperative voice change after thyroidectomy (11, 12). Awan et al. used spectral and cepstral acoustic analyses to track voice change after thyroidectomy and found that sentence-level cepstral measures, especially cepstral peak prominence and its standard deviation, were sensitive to early postoperative deterioration (11) Solomon et al. likewise showed that shimmer, harmonic-to-noise ratio, and cepstral peak prominence tracked postoperative change, with cepstral peak prominence showing the strongest effect (12). Because preoperative voice status varies substantially across individuals, it is often more informative to evaluate postoperative change relative to baseline than to rely on postoperative values alone (11, 12).

What remains unclear is whether early postoperative voice change follows reproducible objective patterns rather than a single uniform course. Most previous studies have treated post-thyroidectomy dysphonia as one entity or have compared predefined groups, but this approach may overlook heterogeneity within early postoperative voice outcomes (13). Unsupervised clustering provides a data-driven approach to identify natural groupings in multidimensional feature space. In the present study, we used partitioning around medoids with Manhattan distance and silhouette-based cluster-number selection, together with principal component analysis for within-subgroup refinement, to explore whether early objective voice change contains clinically meaningful substructure (14–16).

The clinical question, however, is not only whether clusters can be identified, but whether they are associated with outcomes that matter to patients. In this study, we focused on three clinically relevant questions. First, do early postoperative objective voice changes show reproducible data-driven patterns? Second, are some of these patterns associated with clinically important short-term deterioration in patient-reported voice handicap, defined here as a postoperative increase in VHI-30 of at least 13 points (17)? Third, is the resulting phenotype structure statistically stable and reproducible under complementary robustness analyses, including consensus clustering, the proportion of ambiguous clustering, and resampling-based adjusted Rand index reproducibility (18–20)? Importantly, the aim of the present study was to evaluate the association between early objective acoustic patterns and short-term patient-reported and perceptual outcomes. It was not designed to determine whether these patterns were driven predominantly by neural or non-neural mechanisms, nor to determine whether they represent persistent long-term voice phenotypes.

## Methods

### Study design and ethics

This was a single-center, prospective cohort study conducted in the Department of Thyroid Surgery, China-Japan Union Hospital of Jilin University. Consecutive screening and enrollment of eligible patients were performed from October 2025 to November 2025. The study aimed to identify data-driven phenotypes of early postoperative objective voice change and to evaluate their short-term clinical relevance. The protocol was approved by the Ethics Committee of China-Japan Union Hospital of Jilin University before enrollment began (Approval No. 2025091811), and written informed consent was obtained from all participants before any study-specific procedure.

### Participants and cohort flow

Patients undergoing thyroid surgery during the study period were screened consecutively according to prespecified criteria. Eligible participants were adults aged 18 years or older who provided written informed consent, had normal bilateral vocal fold mobility on preoperative laryngoscopy with no clinically relevant laryngeal structural pathology affecting phonation, and were able to complete the standardized voice-recording and clinical assessment protocol. Exclusion criteria included reoperative thyroid surgery; preoperative vocal fold motion impairment or clinically relevant laryngeal structural pathology affecting phonation; prior laryngeal surgery or prior head and neck radiotherapy; neurologic or neuromuscular disorders affecting phonation or articulation; and acute upper respiratory infection or acute laryngitis during the peri-assessment period.

**Figure 1:**
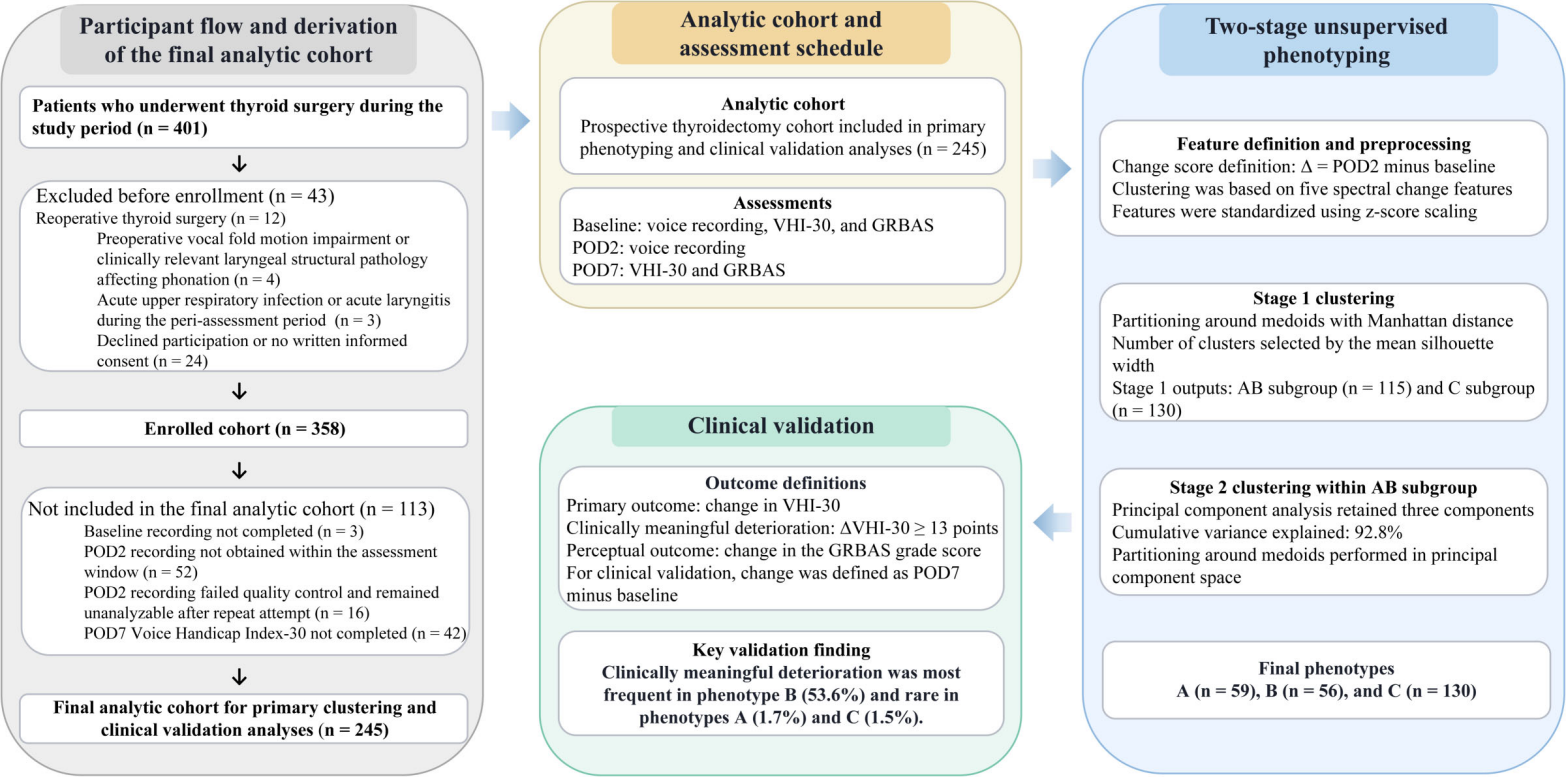
Cohort flow, two-stage phenotyping workflow, and clinical validation framework. The left panel shows cohort flow and final analytic sample formation. The center panels show the analytic cohort and assessment schedule at baseline, POD2, and POD7. The right panel shows the two-stage phenotyping workflow. Stage 1 identified an AB subgroup (n = 115) and a C subgroup (n = 130). Stage 2 further separated the AB subgroup into phenotype A (n = 59) and phenotype B (n = 56). The lower center panel shows the clinical validation endpoints.

Completion of postoperative assessments was not part of the eligibility criteria. All eligible participants were enrolled, and missing follow-up data were handled during analytic cohort formation. As shown in **Figure 1**, 401 patients were screened, 43 were excluded before enrollment, 358 entered the enrolled cohort, and 245 were included in the final analytic cohort for the primary clustering and clinical validation analyses.

### Perioperative management and assessment schedule

All enrolled patients underwent surgery under a standard institutional anesthesia protocol and routine perioperative management. Thyroid procedures were performed by senior thyroid surgeons at the study center.

Assessments were performed at three time points: baseline before surgery, postoperative day 2 (POD2), and postoperative day 7 (POD7). Baseline assessment included standardized voice recording and clinical voice evaluation. POD2 assessment consisted of repeat voice recording for objective feature extraction and phenotype derivation. POD7 assessment consisted of patient-reported and perceptual clinical validation measures.

Systematic postoperative laryngoscopy was not incorporated into the study protocol. This reflected the study’s focus on non-invasive short-term voice assessment and the fact that routine postoperative laryngoscopic reassessment of all patients was not part of the institutional clinical workflow during the study period. Avoiding additional endoscopic assessment also reduced the examination burden for patients without routine clinical indications for postoperative laryngoscopy. Therefore, postoperative vocal fold mobility was unavailable as a study outcome or covariate. No structured superior laryngeal nerve assessment was included. Accordingly, the present study was designed to examine early objective voice-change patterns and their short-term clinical correlates, rather than to determine whether postoperative abnormalities were caused by recurrent laryngeal nerve injury, superior laryngeal nerve injury, or non-neural perioperative factors. The resulting phenotypes should therefore be interpreted as early postoperative voice-change phenotypes, not as nerve-injury phenotypes.

### Voice recording protocol and quality control

Recordings were obtained in a quiet room with ambient noise controlled at 40 dBA or lower. An AKG C410 headset microphone (AKG Acoustics, Vienna, Austria) was used for all recordings, and recordings were captured on a standard laptop computer. The same microphone, setup, and operating procedure were used across time points. Recordings were saved as uncompressed WAV files with a sampling rate of 44.1 kHz and a 16-bit depth.

The recording protocol included standardized voice tasks, including sustained phonation and connected speech. Audio files were de-identified before review and feature extraction. Quality control included checks for file integrity, task completion, excessive environmental noise, clipping or distortion, and severe interruptions leading to unusable phonation. When feasible, recordings that failed quality control were repeated within the assessment window.

### Clinical voice outcomes

Patient-reported outcome was assessed using Voice Handicap Index-30 (VHI-30) (6, 8). Scores range from 0 to 120, with higher scores indicating greater perceived voice-related handicap. VHI-30 was assessed at baseline and POD7. The primary patient-reported validation outcome was change from baseline to POD7, defined as the POD7 score minus the baseline score. A binary responder endpoint was prespecified *a priori* as worsening of at least 13 points (17).

Perceptual outcome was assessed using the GRBAS scale (21). The primary perceptual validation outcome was change in Grade from baseline to POD7. As a sensitivity outcome, change in the total GRBAS score was also evaluated.

### Perceptual rating procedure

GRBAS ratings were independently performed by two otolaryngologists using de-identified audio recordings. Disagreements were adjudicated by a third otolaryngologist. The adjudicated final ratings were used for analysis.

### Objective feature extraction

Objective voice features were extracted using script-based Python pipelines. Two predefined feature sets were used: a core spectral feature set for clustering and a conventional acoustic feature set for phenotype profiling and interpretation. Conventional acoustic features included fundamental frequency, jitter, shimmer, harmonics-to-noise ratio, and maximum phonation time, derived from stable non-silent segments. These variables were not used as clustering inputs (10).

The core clustering inputs were five POD2 spectral change features relative to baseline: root mean square energy, zero-crossing rate, spectral centroid, spectral bandwidth, and a mel-frequency cepstral coefficient summary metric (11, 12). For most spectral features, change was defined as conventional percent change from baseline to POD2. For the mel-frequency cepstral coefficient summary metric, a symmetric percent-change approach was used to improve numerical stability near zero. Detailed extraction parameters are provided in the Supplementary Methods.

Root mean square energy was retained as a prespecified spectral input in the primary analysis and was interpreted within the standardized headset-microphone recording protocol used across time points.

### Data preprocessing and analytic cohort definition

The unit of analysis was the participant. Baseline, POD2, and POD7 data were linked using a de-identified study identifier. A complete-case strategy was used for the core clustering features and primary POD7 validation outcomes.

The five POD2 spectral change features used for clustering were standardized using z-score transformation. Standardization was applied only to clustering features. POD7 clinical outcomes were not used in feature scaling, cluster-number selection, or phenotype derivation.

### Two-stage unsupervised phenotyping

Phenotyping used a prespecified two-stage unsupervised clustering workflow based on partitioning around medoids with Manhattan distance (14). The two-stage strategy was chosen to reflect the expected hierarchical structure of early postoperative objective voice change: the first stage was intended to identify the dominant separation in the full analytic cohort, and the second stage was intended to refine a secondary structure within the subgroup that remained heterogeneous after the first-stage split. This approach was used to avoid forcing all participants into a single flat clustering solution when the data structure suggested a major split followed by within-subgroup refinement.

Stage 1 clustering was performed in the full analytic cohort using the five standardized POD2 spectral change features. Candidate cluster numbers were evaluated over a prespecified range, and mean silhouette width was used as the primary cluster-number selection criterion (15). This stage yielded an AB subgroup and a C subgroup.

Stage 2 clustering was then performed only within the AB subgroup. Principal component analysis was applied to the same feature set within the AB subgroup, and the first three principal components were retained for clustering in principal component space (16). Partitioning around medoids was then re-applied, yielding final phenotypes A and B within the AB subgroup. Together with the Stage 1 C subgroup, this produced the final three-phenotype structure.

POD7 patient-reported and perceptual outcomes were not used for clustering, cluster-number selection, principal component analysis, or phenotype assignment. These clinical outcomes were reserved for subsequent validation of the derived phenotypes.

### Robustness and stability analyses

Robustness was assessed using several prespecified complementary analyses. Consensus clustering was used to evaluate stability under repeated subsampling and reclustering (18), and the proportion of ambiguous clustering was calculated under prespecified ambiguity intervals (19). Sample-level stability was assessed using within-cluster mean consensus and a separation score derived from the consensus matrix. Resampling reproducibility was quantified using the adjusted Rand index.

To address the possibility that a one-step clustering solution might provide a comparable or superior alternative, the primary two-stage workflow was compared with one-step clustering strategies using the same five standardized POD2 spectral change features (20). A one-step k = 3 strategy was evaluated as a flat three-cluster alternative to the final three-phenotype solution. A one-step k = 4 strategy was additionally evaluated to assess whether a four-cluster solution improved cluster separation or resampling reproducibility. These comparisons included final-label mean silhouette width, proportion of ambiguous clustering, and resampling-based adjusted Rand index. A structured summary of the cluster-selection procedure, stability analyses, reproducibility metrics, and related statistical settings is provided in **Supplementary Table 1**.

### Statistical analysis

Continuous variables are presented as median [interquartile range], and categorical variables as number and percentage. Continuous variables were compared using the Kruskal-Wallis test, and categorical variables were compared using the Pearson chi-square test or Fisher exact test, as appropriate. All tests were two-sided.

Overall between-phenotype differences in objective features were tested using Kruskal-Wallis tests, with epsilon-squared reported as the effect size for continuous outcomes. Pairwise comparisons used Wilcoxon rank-sum tests with Benjamini-Hochberg adjustment. Cliff’s delta was reported as the pairwise effect size where applicable.

Clinical validation outcomes included change in VHI-30, the binary VHI-30 responder endpoint, and change in Grade within the GRBAS scale. Continuous outcomes were compared across phenotypes using Kruskal-Wallis tests with epsilon-squared effect size. The responder outcome was compared using the Pearson chi-square test or Fisher exact test, with Cramér’s V reported as the effect size. For responder-rate visualization, exact 95% binomial confidence intervals were calculated using the Clopper-Pearson method.

Additional exploratory analyses were performed to address potential surgical heterogeneity and to evaluate whether demographic, imaging, or operative variables were associated with POD7 clinical outcomes. Operative variables compared across phenotypes included extent of thyroidectomy, surgical approach, central neck dissection, lateral neck dissection, and operative time. Associations between demographic, imaging, and operative variables and POD7 outcomes were evaluated for change in VHI-30, VHI-30 responder status, and change in Grade within the GRBAS scale. For continuous clinical outcomes, continuous predictors were assessed using Spearman correlation, and categorical predictors were assessed using Wilcoxon rank-sum tests or Kruskal-Wallis tests, as appropriate. For the binary responder endpoint, continuous predictors were compared using Wilcoxon rank-sum tests, and categorical predictors were compared using Pearson chi-square or Fisher exact tests, as appropriate. These analyses were considered exploratory, and no multivariable causal model was prespecified.

### Software

Statistical analyses and figure/table generation were performed in R version 4.4.2 (R Foundation for Statistical Computing, Vienna, Austria). Objective feature extraction was performed in Python. Conventional acoustic extraction used Praat via Parselmouth, and spectral feature extraction used librosa with supporting libraries including NumPy and pandas.

## Results

### Cohort flow and final analytic sample formation

During the study period, 401 patients undergoing thyroid surgery were screened for eligibility. Before enrollment, 43 were excluded because of reoperative thyroid surgery (n = 12), preoperative vocal fold motion impairment or clinically relevant laryngeal structural pathology affecting phonation (n = 4), acute upper respiratory infection or acute laryngitis during the peri-assessment period (n = 3), or refusal to participate or lack of written informed consent (n = 24). The enrolled cohort therefore comprised 358 participants. Of these, 113 were not included in the final analytic cohort because baseline recording was not completed (n = 3), POD2 recording was not obtained within the assessment window (n = 52), POD2 recording failed quality control and remained unanalyzable after repeat attempt (n = 16), or POD7 VHI-30 was not completed (n = 42). The final analytic cohort for the primary clustering and clinical validation analyses comprised 245 participants (**Figure 1**).

### Baseline characteristics by phenotype

Baseline characteristics stratified by the final phenotypes are summarized in **Table 1**. In the final analytic cohort, the median age was 47.0 years (interquartile range, 37.0–54.0), 75.9% of participants were female, and the median body mass index was 24.8 kg/m² (interquartile range, 22.3–28.3). Most baseline demographic, laboratory, imaging, and operative variables were broadly comparable across phenotypes. Among the prespecified baseline variables, only central neck dissection differed significantly across groups (P = 0.035), with proportions of 98.3% in phenotype A, 92.9% in phenotype B, and 86.9% in phenotype C.

**Table 1:** Baseline characteristics by final phenotype.

Variable	Overall (N=245)	Phenotype A (n=59)	Phenotype B (n=56)	Phenotype C (n=130)	*P* value
Age, years	47.0 [37.0, 54.0]	44.0 [34.5, 55.0]	46.0 [37.0, 53.3]	47.5 [40.0, 54.0]	.300
Sex, n (%)					.138
Female	186 (75.9%)	43 (72.9%)	38 (67.9%)	105 (80.8%)	
Male	59 (24.1%)	16 (27.1%)	18 (32.1%)	25 (19.2%)	
BMI, kg/m²	24.8 [22.3, 28.3]	24.8 [23.0, 28.3]	24.6 [22.2, 27.9]	24.8 [22.5, 28.3]	.774
Hypertension, n (%)					.755
No	204 (83.3%)	51 (86.4%)	46 (82.1%)	107 (82.3%)	
Yes	41 (16.7%)	8 (13.6%)	10 (17.9%)	23 (17.7%)	
Diabetes mellitus, n (%)					.558
No	223 (91.0%)	53 (89.8%)	53 (94.6%)	117 (90.0%)	
Yes	22 (9.0%)	6 (10.2%)	3 (5.4%)	13 (10.0%)	
Smoking, n (%)					.303
No	208 (84.9%)	52 (88.1%)	44 (78.6%)	112 (86.2%)	
Yes	37 (15.1%)	7 (11.9%)	12 (21.4%)	18 (13.8%)	
Alcohol use, n (%)					.173
No	237 (96.7%)	58 (98.3%)	52 (92.9%)	127 (97.7%)	
Yes	8 (3.3%)	1 (1.7%)	4 (7.1%)	3 (2.3%)	
TSH, mIU/L	2.00 [1.39, 2.89]	2.00 [1.27, 3.02]	1.93 [1.37, 2.67]	2.15 [1.45, 2.92]	.501
FT3, pmol/L	4.81 [4.43, 5.27]	4.97 [4.57, 5.37]	4.95 [4.62, 5.29]	4.72 [4.33, 5.21]	.115
FT4, pmol/L	16.40 [14.76, 17.90]	16.35 [14.97, 17.52]	16.57 [14.80, 18.30]	16.15 [14.70, 17.92]	.682
Maximum nodule diameter on ultrasonography, cm	0.99 [0.65, 1.75]	1.02 [0.62, 1.73]	0.99 [0.72, 1.71]	0.99 [0.64, 1.77]	.979
Posteriorly located nodule on ultrasonography, n (%)					.179
No	164 (66.9%)	45 (76.3%)	34 (60.7%)	85 (65.4%)	
Yes	81 (33.1%)	14 (23.7%)	22 (39.3%)	45 (34.6%)	
Trachea-adjacent nodule on ultrasonography, n (%)					.843
No	150 (61.2%)	36 (61.0%)	36 (64.3%)	78 (60.0%)	
Yes	95 (38.8%)	23 (39.0%)	20 (35.7%)	52 (40.0%)	
Hashimoto thyroiditis, n (%)					.184
No	197 (80.4%)	52 (88.1%)	42 (75.0%)	103 (79.2%)	
Yes	48 (19.6%)	7 (11.9%)	14 (25.0%)	27 (20.8%)	
Focality (unifocal/multifocal)					.286
Unifocal	134 (54.7%)	27 (45.8%)	32 (57.1%)	75 (57.7%)	
Multifocal	111 (45.3%)	32 (54.2%)	24 (42.9%)	55 (42.3%)	
Extent of thyroidectomy (unilateral/bilateral)					.843
Unilateral thyroidectomy	145 (59.2%)	34 (57.6%)	35 (62.5%)	76 (58.5%)	
Bilateral thyroidectomy	100 (40.8%)	25 (42.4%)	21 (37.5%)	54 (41.5%)	
Surgical approach (open/endoscopic)					.156
Open	221 (90.2%)	53 (89.8%)	47 (83.9%)	121 (93.1%)	
Endoscopic	24 (9.8%)	6 (10.2%)	9 (16.1%)	9 (6.9%)	
Central neck dissection, n (%)					.035
No	22 (9.0%)	1 (1.7%)	4 (7.1%)	17 (13.1%)	
Yes	223 (91.0%)	58 (98.3%)	52 (92.9%)	113 (86.9%)	
Lateral neck dissection (None/unilateral/bilateral)					.223
None	227 (92.7%)	53 (89.8%)	51 (91.1%)	123 (94.6%)	
Unilateral	15 (6.1%)	6 (10.2%)	3 (5.4%)	6 (4.6%)	
Bilateral	3 (1.2%)	0 (0.0%)	2 (3.6%)	1 (0.8%)	
Operative time, minutes	90 [70, 110]	90 [75, 112]	90 [65, 110]	85 [70, 108]	.591

Baseline demographic, laboratory, imaging, and operative characteristics are presented by phenotype. Continuous variables are reported as median [interquartile range], and categorical variables as n (%). Overall comparisons were performed using the Kruskal-Wallis test for continuous variables and the Pearson chi-square test or Fisher exact test, as appropriate, for categorical variables.

### Derivation of phenotypes

Stage 1 clustering was performed in the full analytic cohort using the five standardized POD2 spectral change features. Using partitioning around medoids with Manhattan distance and silhouette-based cluster-number selection, the optimal Stage 1 solution was k = 2, yielding an AB subgroup (n = 115) and a C subgroup (n = 130).

Stage 2 clustering was then performed only within the AB subgroup. Principal component analysis was applied to the same feature set, and the first three principal components, explaining 92.8% of cumulative variance, were retained for clustering in principal component space. The optimal Stage 2 solution was k = 2, yielding phenotype A (n = 59) and phenotype B (n = 56). Together with the Stage 1 C subgroup, this produced the final three-phenotype structure A/B/C = 59/56/130.

**Figure 2:**
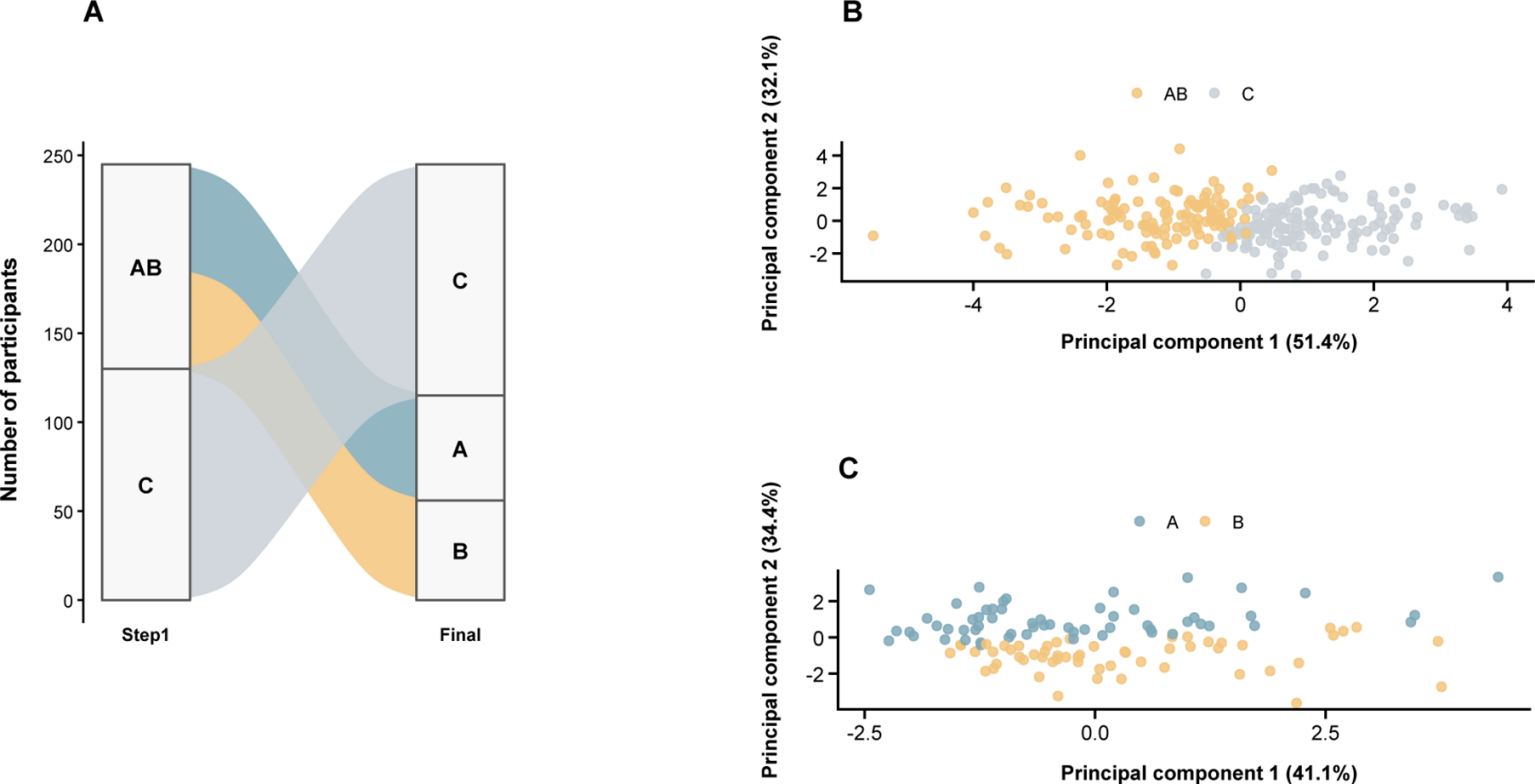
Two-stage clustering structure and final phenotype assignment. The alluvial plot shows the mapping from the Stage 1 grouping (AB vs C) to the final phenotype labels **(A–C)**. The principal component plots show the clustering structure in the full cohort and within the AB subgroup.

The relationship between the Stage 1 grouping and the final phenotype labels is shown in **Figure 2**. The alluvial plot demonstrates that phenotype C was retained directly from Stage 1, whereas phenotypes A and B arose from further subdivision of the AB subgroup. The principal component plots provide complementary low-dimensional visualization of the clustering structure in the full cohort and within the AB subgroup.

### Objective phenotype profiles at POD2

**Figure 3:**
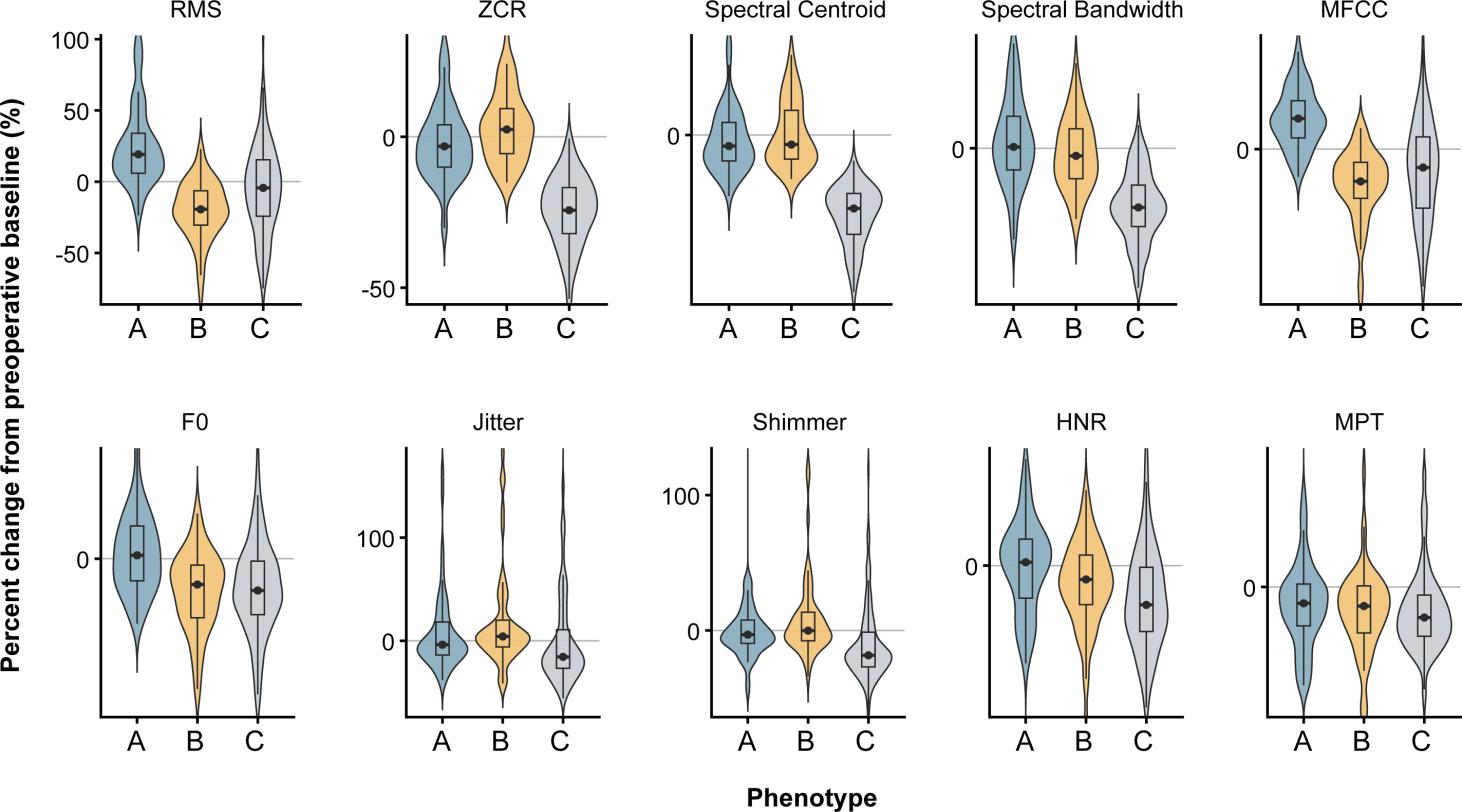
Objective phenotype profiles at POD2. The upper row shows the five spectral change features used as clustering inputs: root mean square energy, zero-crossing rate, spectral centroid, spectral bandwidth, and the mel-frequency cepstral coefficient summary metric. The lower row shows conventional acoustic change features used for phenotype profiling but not for clustering. Violin plots show distributions, box plots show median and interquartile range, and jittered points show individual participants. Change values are relative to baseline.

Objective POD2 voice-change features showed clear differences across the three phenotypes (**Figure 3**; **Table 2**). All five spectral clustering inputs differed significantly across phenotypes (all P < 0.001). The largest overall effects were observed for Delta spectral centroid (epsilon-squared = 0.721) and Delta zero-crossing rate (epsilon-squared = 0.605), followed by Delta spectral bandwidth (epsilon-squared = 0.414). The mel-frequency cepstral coefficient summary metric and root mean square energy also showed substantial between-phenotype differences, with epsilon-squared values of 0.296 and 0.272, respectively.

**Table 2:** POD2 spectral feature changes by phenotype.

Feature (change from baseline, %)	Phenotype A median [IQR]	Phenotype B median [IQR]	Phenotype C median [IQR]	*P* value	ϵ²	*H* statistic
RMS energy	19.14 [5.92, 33.93]	-19.39 [-30.49, -6.43]	-4.36 [-24.26, 15.40]	< .001	0.272	67.76
Zero-crossing rate	-3.17 [-10.05, 3.98]	2.42 [-5.57, 9.33]	-24.33 [-32.06, -16.82]	< .001	0.605	148.39
Spectral centroid	-3.10 [-7.23, 3.49]	-2.69 [-6.73, 6.84]	-20.50 [-27.73, -16.31]	< .001	0.721	176.51
Spectral bandwidth	0.40 [-6.55, 9.68]	-2.29 [-9.19, 5.93]	-17.91 [-23.68, -11.14]	< .001	0.414	102.08
MFCC	6.37 [2.34, 10.08]	-6.71 [-10.25, -2.74]	-3.83 [-12.28, 2.56]	< .001	0.296	73.74
HNR	1.00 [-9.72, 7.86]	-4.14 [-11.62, 3.13]	-11.70 [-19.66, -0.52]	< .001	0.078	20.94
Shimmer	-3.10 [-9.51, 7.63]	-0.14 [-7.64, 13.44]	-18.36 [-26.92, -1.24]	< .001	0.157	40.11

POD2 changes in the spectral features used as clustering inputs are presented by phenotype. Continuous variables are reported as median [interquartile range]. Overall comparisons were performed using the Kruskal-Wallis test, with epsilon-squared reported as the effect size. Pairwise comparisons used Wilcoxon rank-sum tests with Benjamini-Hochberg adjustment.

The phenotype profiles were distinct. Phenotype A showed positive shifts in root mean square energy and the mel-frequency cepstral coefficient summary metric, whereas zero-crossing rate, spectral centroid, and spectral bandwidth remained close to the neutral range overall. Phenotype B showed the opposite direction for root mean square energy and the mel-frequency cepstral coefficient summary metric, with the remaining spectral features again centered near zero. In contrast, phenotype C showed pronounced decreases in zero-crossing rate, spectral centroid, and spectral bandwidth, together with more modest negative shifts in root mean square energy and the mel-frequency cepstral coefficient summary metric.

Conventional acoustic variables were used for phenotype profiling and interpretation but not for clustering. Among these variables, Delta fundamental frequency, Delta jitter, Delta shimmer, and Delta harmonics-to-noise ratio differed significantly across phenotypes, whereas Delta maximum phonation time did not (**Supplementary Table 2**). Among the conventional acoustic measures, Delta shimmer showed the largest effect size.

### Primary clinical validation at POD7

**Figure 4:**
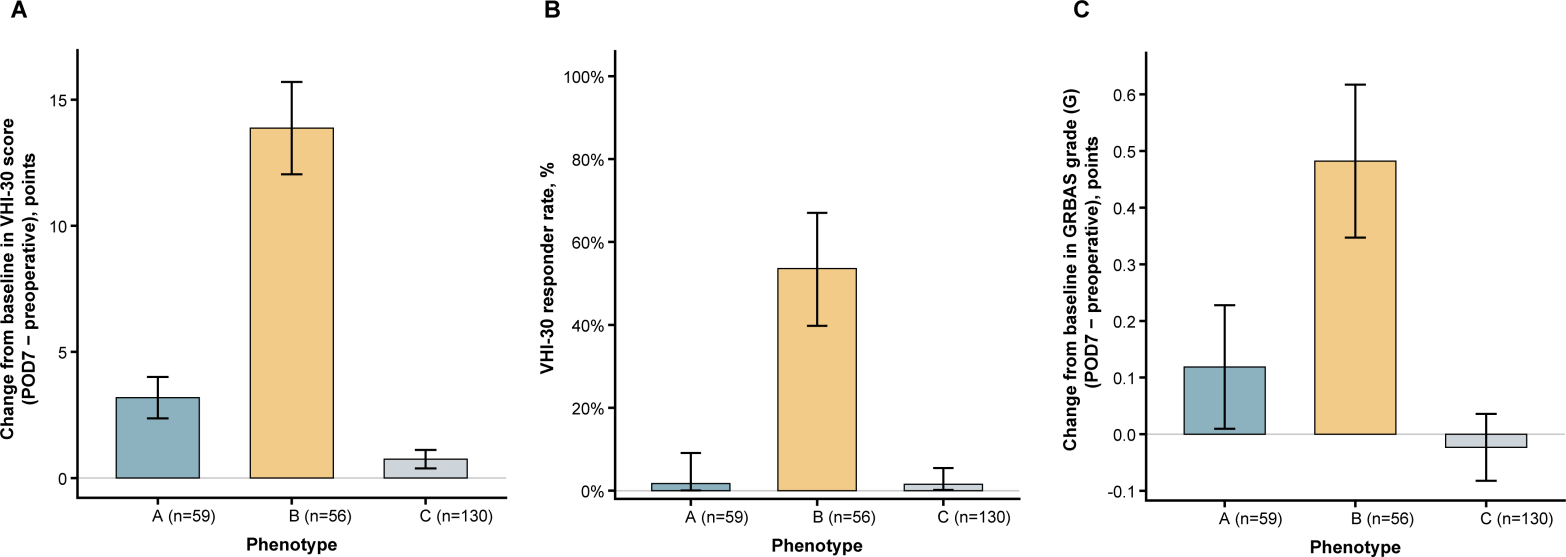
Clinical validation of phenotypes at POD7. Clinical outcomes at POD7 by phenotype (A, n = 59; B, n = 56; C, n = 130). **(A)** Change from baseline in Voice Handicap Index-30 score, shown as violin plots with embedded box plots and jittered individual observations. **(B)** Voice Handicap Index-30 responder rate, defined *a priori* as Delta Voice Handicap Index-30 >= 13 points, shown as point estimates with Exact 95% binomial confidence intervals (Clopper-Pearson). **(C)** Change from baseline in Grade (G) within the Grade, Roughness, Breathiness, Asthenia, Strain scale, shown as violin plots with embedded box plots and jittered individual observations.

Primary POD7 clinical validation outcomes are summarized in **Table 3** and visualized in **Figure 4**. For clinical interpretation, baseline voice-related status was generally similar across groups, although baseline VHI-30 differed modestly across phenotypes (P = 0.025; epsilon-squared = 0.022), whereas baseline Grade showed no significant between-phenotype difference (P = 0.195; epsilon-squared = 0.005).

**Table 3:** Clinical voice outcomes at baseline and POD7 by phenotype.

Outcome	Phenotype A (n=59)	Phenotype B (n=56)	Phenotype C (n=130)	*P* value	Effect size
VHI-30 change from baseline to POD7, median [IQR]	2.0 [2.0, 4.0]	13.0 [9.0, 16.3]	0.0 [0.0, 1.0]	< .001	0.641
VHI-30 responder (change ≥ 13 points), n/N (%)	1/59 (1.7%)	30/56 (53.6%)	2/130 (1.5%)	< .001	0.639
GRBAS grade (G) change from baseline to POD7, median [IQR]	0 [0, 0]	0 [0, 1]	0 [0, 0]	< .001	0.203

Clinical voice outcomes are presented by phenotype for baseline, POD7, and change from baseline to POD7. Continuous outcomes are reported as median [interquartile range], and the responder outcome as n/N (%). Overall comparisons used the Kruskal-Wallis test for continuous outcomes and the Pearson chi-square test or Fisher exact test, as appropriate, for the responder outcome. Effect sizes are reported as epsilon-squared for continuous outcomes and Cramér’s V for the responder outcome.

Phenotypes differed significantly in Delta VHI-30 (P < 0.001; epsilon-squared = 0.641). Median changes were 2.0 [2.0, 4.0] in phenotype A, 13.0 [9.0, 16.3] in phenotype B, and 0.0 [0.0, 1.0] in phenotype C. Pairwise comparisons further supported strong separation of phenotype B from both phenotype A and phenotype C, with Cliff’s delta values of -0.903 (95% confidence interval, -0.966 to -0.824) for A versus B and 0.974 (95% confidence interval, 0.945 to 0.995) for B versus C. The A versus C comparison was also significant, with Cliff’s delta 0.665 (95% confidence interval, 0.522 to 0.797).

Using the prespecified responder definition of Delta VHI-30 ≥ 13, responder rates differed markedly across phenotypes (P < 0.001; Cramér’s V = 0.639). Phenotype B had the highest responder proportion, with 30/56 (53.6%; exact 95% confidence interval, 39.7%–67.0%), compared with 1/59 (1.7%; exact 95% confidence interval, 0.0%–9.1%) in phenotype A and 2/130 (1.5%; exact 95% confidence interval, 0.2%–5.4%) in phenotype C.

Phenotypes also differed significantly in Delta Grade within the GRBAS scale (P < 0.001; epsilon-squared = 0.203). Median changes were 0 [0, 0] in phenotype A, 0 [0, 1] in phenotype B, and 0 [0, 0] in phenotype C. Pairwise effect-size comparisons showed Cliff’s delta values of -0.347 (95% confidence interval, -0.502 to -0.189) for A versus B and 0.472 (95% confidence interval, 0.338 to 0.599) for B versus C; the A versus C comparison was smaller but remained significant, with Cliff’s delta 0.133 (95% confidence interval, 0.025 to 0.244).

As a sensitivity analysis, change in total GRBAS score also differed significantly across phenotypes, with the greatest worsening observed in phenotype B.

### Exploratory operative and clinical-factor analyses

Exploratory operative-stratification analyses are summarized in **Supplementary Table 4**. Thyroidectomy extent, surgical approach, lateral neck dissection, and operative time were comparable across the three phenotypes. Central neck dissection differed modestly across phenotypes (P = 0.035), with proportions of 98.3% in phenotype A, 92.9% in phenotype B, and 86.9% in phenotype C.

Exploratory analyses of demographic, imaging, and operative variables in relation to POD7 clinical outcomes are summarized in **Supplementary Table 5**. Phenotype was strongly associated with Delta VHI-30, VHI-30 responder status, and Delta Grade within the GRBAS scale (all P < 0.001). Most demographic, imaging, and operative variables were not associated with these outcomes. Sex (P = 0.027) and Hashimoto thyroiditis (P = 0.035) showed exploratory associations with VHI-30 responder status, but not with Delta VHI-30 or Delta Grade. No operative variable was significantly associated with VHI-30 responder status.

### Robustness and stability analyses

Consensus-clustering diagnostics supported the stability of the primary phenotype structure (**Supplementary Figure 3**). The consensus matrix showed coherent within-phenotype block patterns, and the proportion of ambiguous clustering values was 0.402 for the 0.1–0.9 interval and 0.251 for the 0.2–0.8 interval.

Sample-level stability analysis identified 23 of 245 samples (9.4%) with separation scores below the prespecified threshold of 0.20, indicating a minority of boundary-like cases (**Supplementary Figure 5**). These were distributed as 7 in phenotype A, 16 in phenotype B, and 0 in phenotype C.

Resampling-based reproducibility comparison showed higher adjusted Rand index values for the primary two-stage workflow than for a one-step k = 3 strategy (**Supplementary Figure 4**). The adjusted Rand index was 0.707 [0.630–0.877] for the primary two-stage workflow and 0.689 [0.328–0.793] for the one-step k = 3 strategy.

Additional sensitivity comparison with one-step clustering alternatives is summarized in **Supplementary Table 3**. The primary two-stage workflow showed a final-label mean silhouette width of 0.231, proportion of ambiguous clustering values of 0.402 for the 0.1–0.9 interval and 0.251 for the 0.2–0.8 interval, and a median adjusted Rand index of 0.707 [0.630–0.877]. The one-step k = 3 strategy showed a final-label mean silhouette width of 0.211, higher proportion of ambiguous clustering values, and a median adjusted Rand index of 0.689 [0.328–0.793]. The one-step k = 4 strategy did not improve reproducibility, with a final-label mean silhouette width of 0.220, proportion of ambiguous clustering values of 0.500 for the 0.1–0.9 interval and 0.353 for the 0.2–0.8 interval, and a lower median adjusted Rand index of 0.436 [0.360–0.528]. These findings did not support replacing the prespecified two-stage workflow with a one-step k = 3 or k = 4 solution.

## Discussion

### Principal findings

This study identified three early postoperative voice-change phenotypes after thyroid surgery using a prespecified two-stage unsupervised clustering workflow based on POD2 objective spectral features. These phenotypes were associated with short-term clinical outcomes at POD7. Phenotype B showed the greatest worsening in VHI-30, the highest responder rate, and less favorable perceptual outcomes. Phenotype C showed the most distinct spectral pattern, but its short-term patient-reported burden was lower than that of phenotype B. Consensus-based and resampling-based analyses supported the stability of the main phenotype structure in this cohort. Additional comparisons with one-step k = 3 and k = 4 clustering did not show better stability or reproducibility than the prespecified two-stage workflow.

The main finding is that early postoperative voice change after thyroid surgery was not a single uniform pattern in this cohort. Patients with different POD2 objective spectral patterns had different POD7 clinical profiles. These results support the value of using objective early voice measurements to describe short-term postoperative voice heterogeneity, but they should not be interpreted as proving fixed long-term categories or specific biological mechanisms.

### Clinical interpretation

A clinically important finding was the mismatch between objective spectral distinctness and short-term subjective burden. Phenotype C had the largest shifts in structural spectral features, especially zero-crossing rate, spectral centroid, and spectral bandwidth. However, phenotype B had the largest increase in patient-reported voice handicap and the highest rate of clinically meaningful deterioration. This means that the most visually or acoustically distinct pattern was not necessarily the pattern with the highest perceived voice burden.

This finding is clinically relevant because postoperative voice assessment should not rely on a single objective parameter. A patient may have clear acoustic changes without the greatest subjective burden, while another patient may report more obvious functional impairment despite less pronounced changes in some spectral dimensions. The strong separation in VHI-30 change and responder rates suggests that the clustering results were not only mathematical groupings, but were linked to outcomes that patients could perceive.

The perceptual findings were consistent with the patient-reported results, although the magnitude of Grade change was modest. Phenotype B showed a heavier perceptual burden at the distribution level, supporting its interpretation as the less favorable short-term clinical phenotype. However, the perceptual results should be interpreted as supportive evidence rather than the sole basis for clinical interpretation.

The exploratory operative and clinical-factor analyses also help interpret the phenotype results. Thyroidectomy extent, surgical approach, lateral neck dissection, and operative time were broadly comparable across phenotypes. Central neck dissection differed modestly across groups, but no operative variable was significantly associated with VHI-30 responder status. Therefore, the worse short-term clinical profile of phenotype B was not simply explained by more extensive surgery or by a specific surgical approach. Sex and Hashimoto thyroiditis showed exploratory associations with VHI-30 responder status, but these findings should be interpreted cautiously because the analyses were exploratory and were not based on a prespecified multivariable causal model.

### Relation to current understanding of post-thyroidectomy voice change

The present findings are consistent with previous work showing that voice change after thyroid surgery is multifactorial and cannot be explained only by overt recurrent laryngeal nerve injury (4, 22–25). A systematic review and meta-analysis found that several acoustic voice parameters worsened in the early postoperative period after uncomplicated thyroidectomy (22). Earlier clinical work also showed that voice changes may occur despite preserved recurrent laryngeal nerve function (23). More recent reviews and prospective studies have similarly emphasized that postoperative voice impairment can occur with intact laryngeal nerve function and may reflect both neural and non-neural factors (24, 25). The present results fit this clinical background, but they do not identify the exact cause of each phenotype.

The timing of the assessments is important. In this study, phenotypes were derived from POD2 recordings and clinically validated using POD7 outcomes. These are early postoperative time points. Voice changes during this period may be affected by tissue edema, intubation-related irritation, temporary neuromuscular effects, pain, reduced willingness to phonate, or protective voice use. Therefore, the phenotypes identified here should be understood as early postoperative voice-change patterns. They should not be assumed to represent persistent voice dysfunction. Additional recordings after early recovery, especially at 1 to 3 months and 6 months, would be needed to determine whether these early patterns resolve, merge, or remain clinically relevant over time.

This distinction is especially important because systematic postoperative laryngoscopy was not included in the study protocol. Postoperative vocal fold mobility was therefore unavailable as an outcome or covariate. No structured superior laryngeal nerve assessment was performed. As a result, the study could not determine whether the observed voice changes were related to recurrent laryngeal nerve injury, superior laryngeal nerve dysfunction, intubation effects, local edema, or other non-neural perioperative factors. The phenotypes should therefore be interpreted as early postoperative voice-change phenotypes, not as nerve-injury phenotypes.

### Why multidomain evaluation matters

The findings also support the need for multidomain voice evaluation after thyroid surgery. VHI-30, perceptual Grade, and objective spectral or acoustic features did not provide identical information (4, 9, 24). Patient-reported handicap reflects the patient’s perceived functional burden. Perceptual ratings reflect clinician-assessed voice quality. Objective features describe measurable properties of the voice signal. These domains overlap, but none can fully replace the others.

This helps explain why phenotype C could have a distinct objective spectral profile without the highest short-term patient-reported burden, and why phenotype B could show the highest clinical burden despite being less distinct in some structural spectral features. In clinical practice, objective early phenotyping may therefore be useful as an additional layer of assessment, but it should be interpreted together with patient-reported and perceptual measures.

### Methodological considerations

Several methodological points should be considered when interpreting the results. Baseline VHI-30 differed modestly across phenotypes, whereas baseline Grade was similar across groups. The larger differences observed at POD7 and in change from baseline suggest that the phenotype structure was linked to short-term postoperative voice burden rather than only reflecting baseline differences.

The two-stage clustering workflow also requires explanation. The workflow was prespecified to first identify the main structure in the full cohort and then further separate the AB subgroup, which remained heterogeneous after the first split. This was done to avoid forcing all patients into a single flat clustering solution when the data suggested a larger split followed by a smaller subgroup structure. POD7 outcomes were not used for clustering, cluster-number selection, principal component analysis, or phenotype assignment. This separation between phenotype derivation and clinical validation helped reduce the risk that the clusters were driven by the clinical outcomes themselves.

Sensitivity analyses were added to examine whether a simpler one-step solution would be preferable. The one-step k = 4 solution did not improve reproducibility and had a lower median adjusted Rand index than the primary workflow. The one-step alternatives also had higher proportions of ambiguous clustering than the primary workflow. These results support retaining the prespecified two-stage workflow for this dataset. Even so, clustering results from a single cohort should not be treated as final. Independent validation is still needed.

The A/B separation was mainly related to energy- and cepstral-related changes, whereas the C pattern was driven more by non-energy spectral features. Root mean square energy was retained as a prespecified feature and was interpreted within the standardized headset-microphone recording protocol used across all time points. For this reason, the phenotypes should be interpreted at the overall pattern level rather than by overinterpreting any single feature.

Perceptual voice assessment was based on final GRBAS ratings from two independent otolaryngologists with third-rater adjudication. Because the perceptual changes were modest, these results should be viewed as supportive evidence. The main clinical signal was strongest for VHI-30 change and the responder endpoint.

### Strengths

This study has several strengths. It used a prospective cohort design with prespecified assessment time points. Phenotype derivation was separated from POD7 clinical validation. The analysis included consensus diagnostics, sample-level stability assessment, and resampling reproducibility. The revised analyses also compared the primary workflow with one-step k = 3 and k = 4 alternatives, directly addressing whether a flat clustering solution would perform better. In addition, exploratory analyses of operative, demographic, and imaging variables helped show that the main clinical phenotype difference was not simply explained by surgical approach or operative extent. The cohort flow was also reported transparently, and follow-up completion was handled during analytic cohort formation rather than being treated as an eligibility criterion.

### Limitations and future directions

Several limitations should be emphasized. First, this was a single-center study, and the phenotypes were derived and evaluated in the same cohort. Surgical technique, perioperative management, recording conditions, and patient characteristics may differ across centers. The phenotype structure therefore needs external validation before it can be used more broadly.

Second, follow-up was short. The study assessed POD2 objective changes and POD7 clinical outcomes. These early postoperative findings may be influenced by transient factors such as edema, intubation irritation, temporary neuromuscular effects, pain, and protective voice use. The study cannot determine whether the phenotypes persisted, resolved, or changed after the first postoperative week. Future studies should include repeated recordings after recovery, preferably at 1 to 3 months and 6 months, to determine whether early phenotypes are associated with persistent voice handicap or delayed recovery.

Third, systematic postoperative laryngoscopy was not performed, and no structured superior laryngeal nerve assessment was included. Therefore, postoperative vocal fold mobility was unavailable, and the study could not separate recurrent laryngeal nerve injury, superior laryngeal nerve dysfunction, and non-neural perioperative causes. This is a major limitation for mechanism interpretation. The identified groups should be described as early objective voice-change patterns with short-term clinical correlates, not as nerve-injury phenotypes.

Fourth, the exploratory analyses of demographic, imaging, and operative variables were not designed to identify independent predictors or causal effects. Sex and Hashimoto thyroiditis showed exploratory associations with VHI-30 responder status, but the number of responder events was limited and no multivariable causal model was prespecified. These findings should be tested in larger datasets.

Future studies should focus on three practical next steps. First, the three-phenotype structure should be tested in independent cohorts, preferably from multiple centers. Second, longer follow-up with repeated voice recordings should be used to determine whether early patterns predict recovery or persistent symptoms. Third, future protocols should add postoperative laryngoscopy, structured superior laryngeal nerve assessment, and more detailed perioperative data so that the sources of postoperative voice changes can be examined more directly.

## Conclusion

In this prospective thyroid surgery cohort, a prespecified two-stage unsupervised clustering workflow identified three early postoperative objective voice-change phenotypes with distinct short-term clinical profiles. Among them, phenotype B showed the greatest patient-reported voice burden and the highest rate of clinically meaningful deterioration at POD7. These findings suggest that early postoperative voice change after thyroid surgery is heterogeneous and can be organized into clinically interpretable short-term patterns. However, these phenotypes should be interpreted as early postoperative voice-change patterns rather than as nerve-injury phenotypes or persistent long-term voice categories.

## Data Availability

The raw data supporting the conclusions of this article will be made available by the authors, without undue reservation.

## Supplementary material

Supplementary Figure 1 Stage 1 cluster-number diagnostics in the full cohort. Cluster-number diagnostics for Stage 1 using the five standardized POD2 spectral change features in the full analytic cohort. Mean silhouette width was used as the primary selection criterion.

Supplementary Figure 2 Stage 2 cluster-number diagnostics within the AB subgroup. Cluster-number diagnostics for Stage 2 within the AB subgroup. Clustering was performed in principal component space. The first three principal components explained 92.8% of cumulative variance.

Supplementary Figure 3 Consensus clustering diagnostics. Consensus matrix heatmap and cumulative distribution function curves from repeated subsampling and reclustering. Proportion of ambiguous clustering values under the prespecified ambiguity intervals (0.1–0.9 and 0.2–0.8) are shown.

Supplementary Figure 4 Resampling-based reproducibility of the two-stage workflow and a one-step three-cluster strategy. Agreement between resampled labels and the full-sample reference labels was quantified using the adjusted Rand index.

Supplementary Figure 5 Sample-level stability and boundary-case assessment. Sample-level stability derived from the consensus matrix is shown using within-cluster mean consensus and a separation score. Samples meeting the prespecified criteria were classified as core samples; lower separation scores indicate more ambiguous assignment.
