# Supplementary figures and images for: Early postoperative voice-change phenotypes after thyroid surgery: a prospective cohort study
Source: Front Endocrinol (Lausanne). 2026 Jun 15;17:1845546. doi: 10.3389/fendo.2026.1845546 (PMC13310725; doi:10.3389/fendo.2026.1845546)

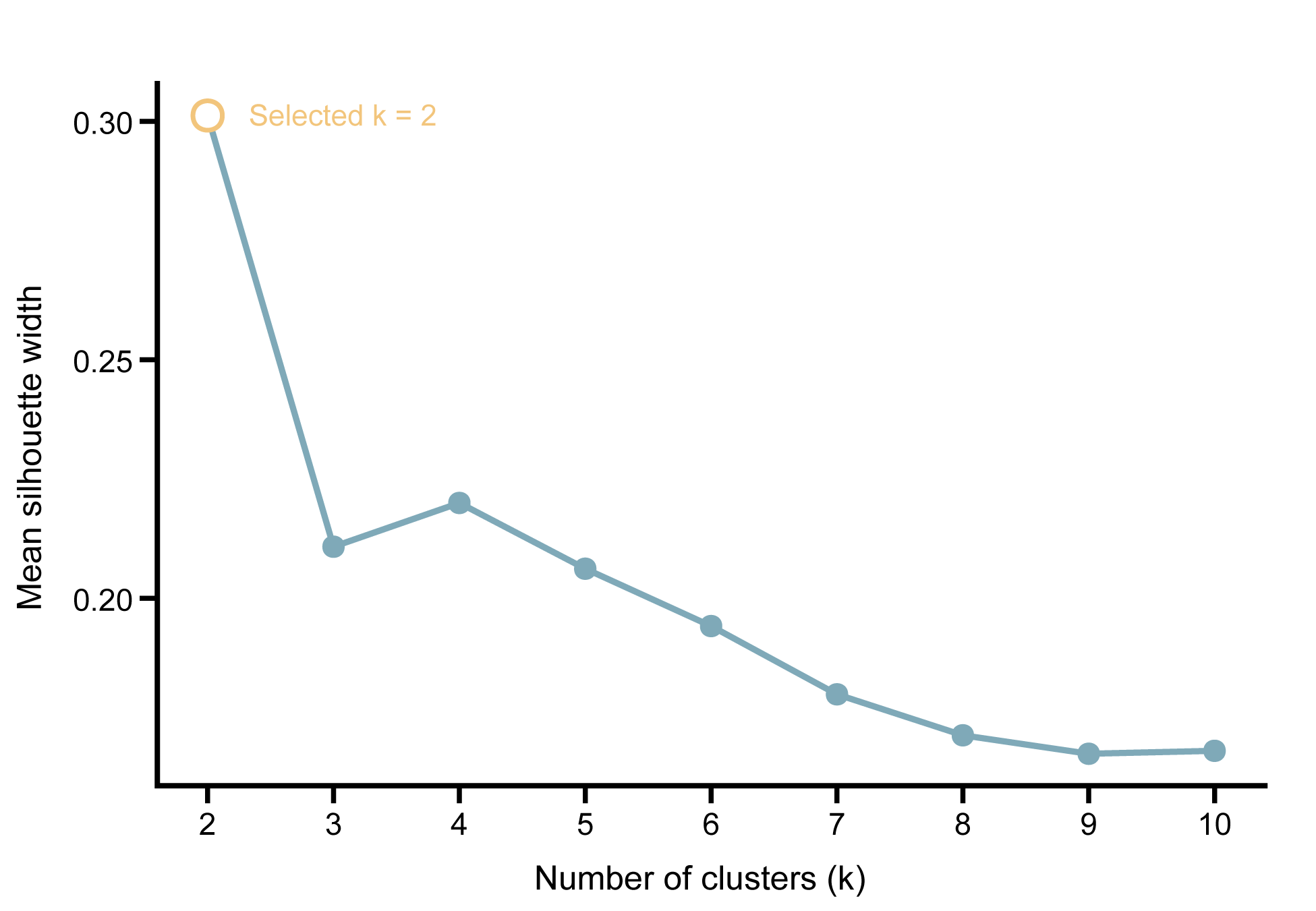

Supplement: Supplementary Figure 1 — Stage 1 cluster-number diagnostics in the full cohort. Cluster-number diagnostics for Stage 1 using the five standardized POD2 spectral change features in the full analytic cohort. Mean silhouette width was used as the primary selection criterion. [file SupplementaryFile1.tif]

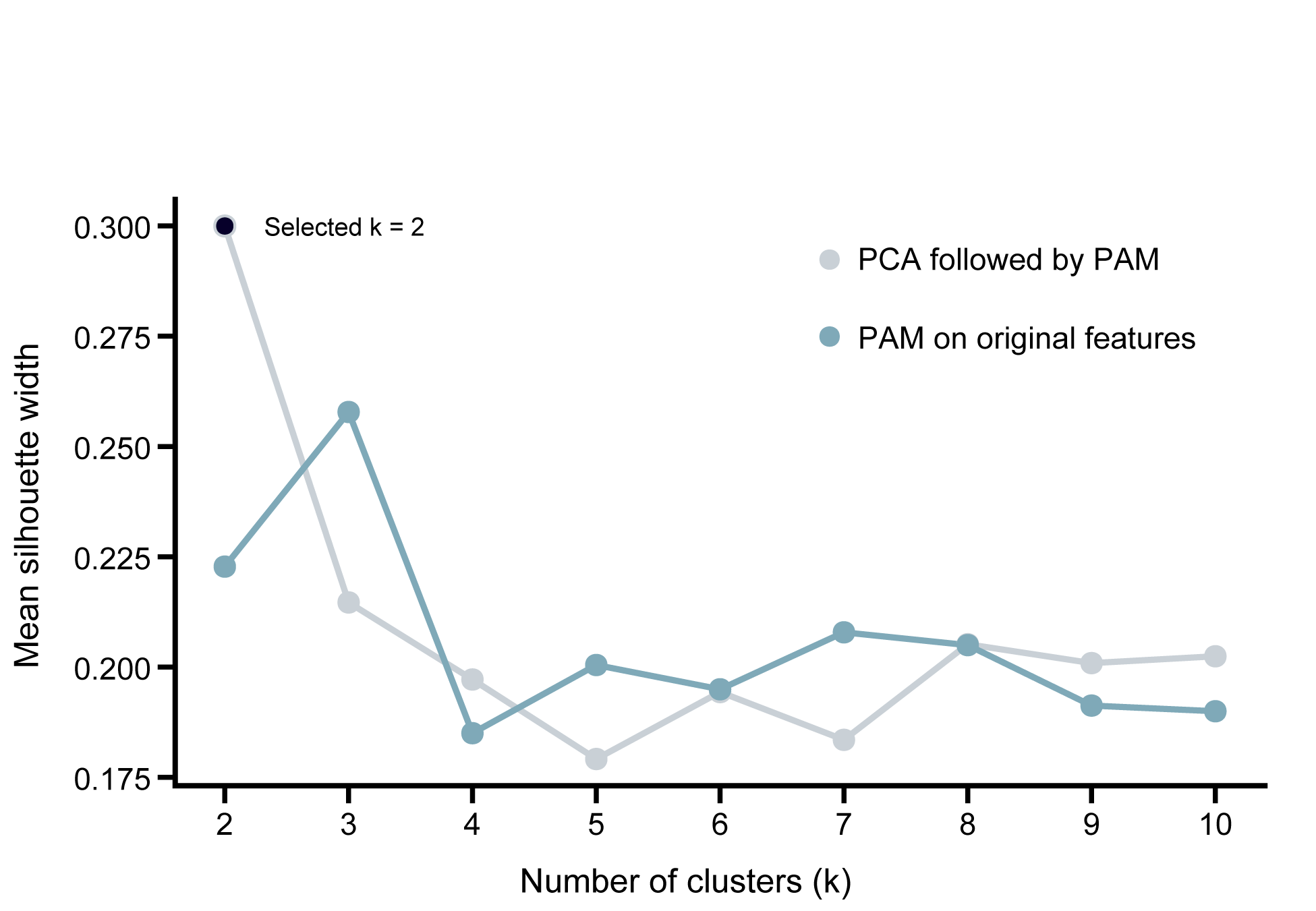

Supplement: Supplementary Figure 2 — Stage 2 cluster-number diagnostics within the AB subgroup. Cluster-number diagnostics for Stage 2 within the AB subgroup. Clustering was performed in principal component space. The first three principal components explained 92.8% of cumulative variance. [file SupplementaryFile2.tif]

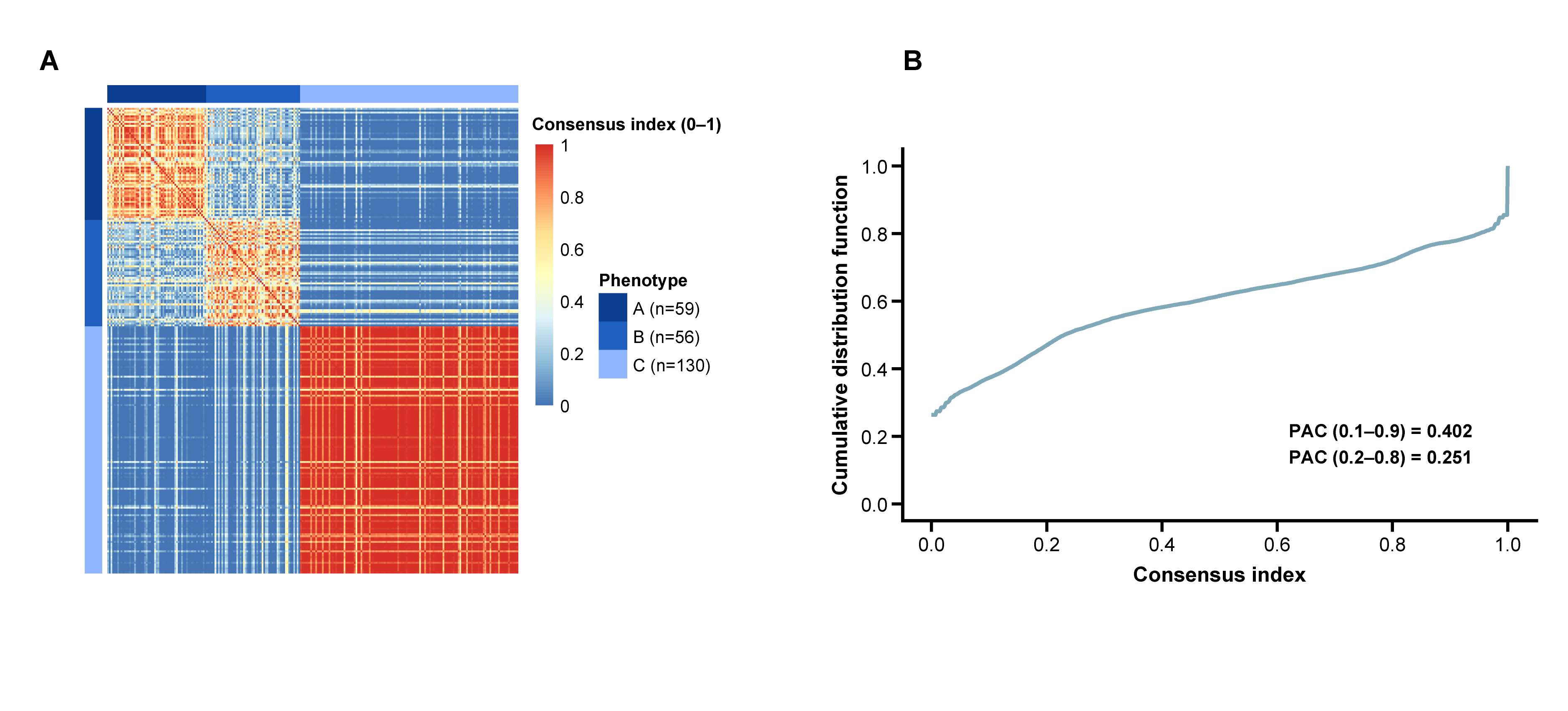

Supplement: Supplementary Figure 3 — Consensus clustering diagnostics. Consensus matrix heatmap and cumulative distribution function curves from repeated subsampling and reclustering. Proportion of ambiguous clustering values under the prespecified ambiguity intervals (0.1–0.9 and 0.2–0.8) are shown. [file SupplementaryFile3.tif]

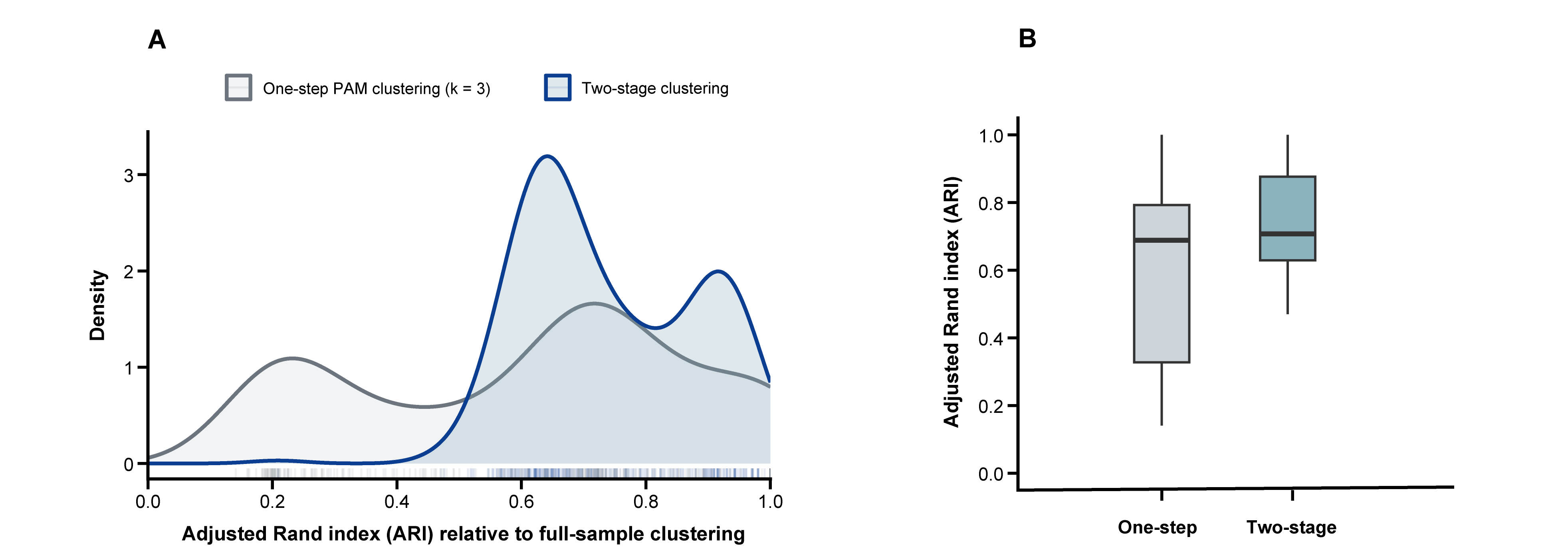

Supplement: Supplementary Figure 4 — Resampling-based reproducibility of the two-stage workflow and a one-step three-cluster strategy. Agreement between resampled labels and the full-sample reference labels was quantified using the adjusted Rand index. [file SupplementaryFile4.tif]

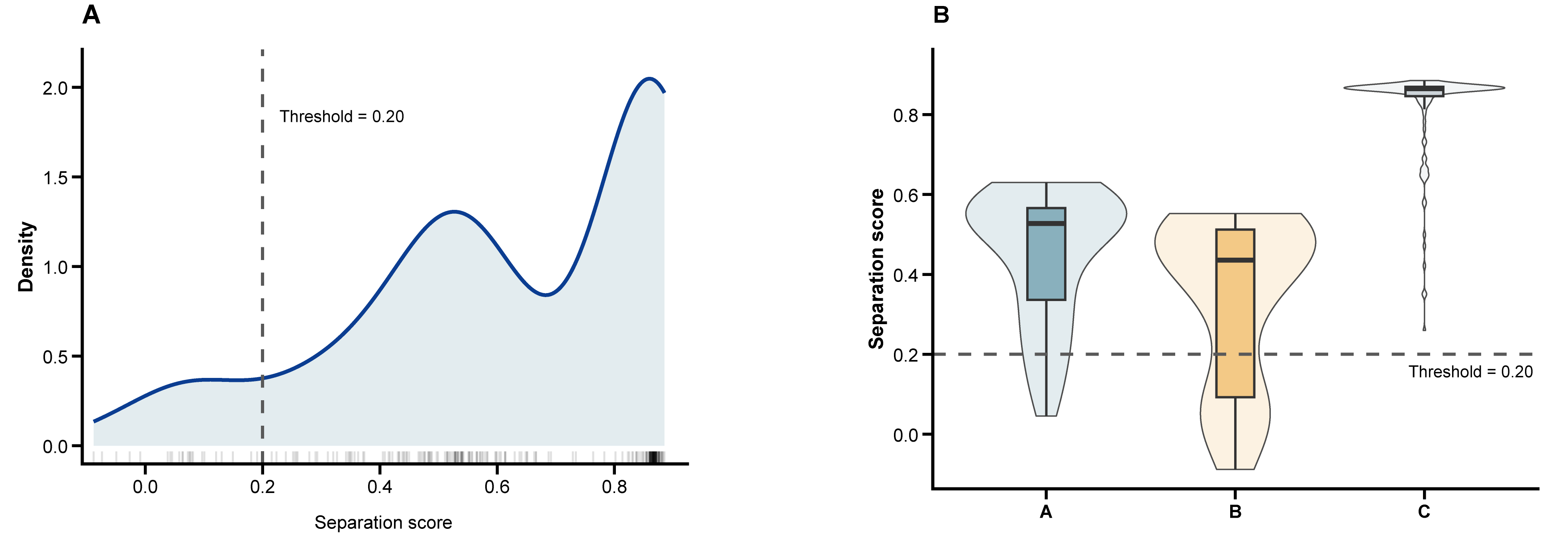

Supplement: Supplementary Figure 5 — Sample-level stability and boundary-case assessment. Sample-level stability derived from the consensus matrix is shown using within-cluster mean consensus and a separation score. Samples meeting the prespecified criteria were classified as core samples; lower separation scores indicate more ambiguous assignment. [file SupplementaryFile5.tif]
