# Supplementary material for: Early postoperative voice-change phenotypes after thyroid surgery: a prospective cohort study
Source: Front Endocrinol (Lausanne). 2026 Jun 15;17:1845546. doi: 10.3389/fendo.2026.1845546 (PMC13310725; doi:10.3389/fendo.2026.1845546)
Supplement: Supplementary file 6 [file Table1.docx]

Table S1

| **Module** | **Item** | **Setting** | **Notes** |
| --- | --- | --- | --- |
| Cohort & timepoints | Sample size | N=245 (complete cases) | Complete-case for clustering features. |
| Cohort & timepoints | Objective features time window | Baseline to postoperative day 2 (change features) | Phenotypes defined using objective change features at postoperative day 2 (POD2). |
| Feature engineering | Spectral Δ features | ΔRMS energy (%), Δzero-crossing rate (%), Δspectral centroid (%), Δspectral bandwidth (%), ΔMFCC (symmetric) | Four traditional spectral features plus one symmetric Mel-frequency cepstral coefficient feature. |
| Preprocessing | Scaling | Global z-score standardization | Mean and standard deviation estimated from the full cohort and reused for all stability/consensus runs. |
| Distance | Metric | Manhattan | Robust for Δ features; used in PAM. |
| Step1 clustering | Algorithm | Partitioning around medoids (PAM), k = 2 | k selected by mean silhouette width in the full cohort. |
| Step1 clustering | Output | AB vs C | Major split. |
| Step2 clustering | Scope | Within AB only | Step2 not applied to C. |
| Step2 clustering | Dimension reduction | Principal component analysis (PCA) within AB subgroup | Three principal components retained; cumulative variance explained reported. |
| Step2 clustering | Algorithm / k | PAM on PC scores, k = 2 (primary) | k = 3 evaluated as a sensitivity analysis, if reported. |
| Final phenotype | Counts | A=59, B=56, C=130 | Final labels from two-stage pipeline. |
| Robustness (consensus) | Iterations / fraction | 200 iterations; 80% subsampling per iteration | Consensus matrix + CDF + PAC. |
| Robustness (consensus) | PAC | PAC (0.1–0.9) = 0.402; PAC (0.2–0.8) = 0.251 | Lower proportion of ambiguous clustering (PAC) indicates higher stability. |
| Robustness (sample-level) | Core definition | Within-phenotype mean consensus ≥ 0.70 and separation score ≥ 0.20 | Core vs non-core samples; lowest-20 separation scores listed separately. |
| Robustness (sample-level) | Core counts | A 37/59; B 15/56; C 126/130 | C highly stable; B more borderline. |
| Robustness (subsampling ARI) | Runs / fraction | 300 runs; 80% subsampling per run | ARI vs full-data reference labels. |
| Robustness (subsampling ARI) | ARI summary | Two-stage: median 0.707 (IQR 0.247); one-step: median 0.689 (IQR 0.465) | Adjusted Rand index (ARI) computed relative to full-sample reference labels; two-stage reduces variability. |
| Stats (phenotype profiles) | Tests | Kruskal–Wallis test + ε²; pairwise Wilcoxon rank-sum (BH) + Cliff’s δ | Nonparametric comparisons across phenotypes with effect sizes and multiple-testing correction. |
| Clinical outcomes | Endpoints | Change in VHI-30 from baseline to POD7; responder (change ≥ 13); change in GRBAS grade G from baseline to POD7 | POD7, postoperative day 7. GRBAS: grade G as primary; total score as sensitivity outcome. |
| Software | R / packages | R + readxl/dplyr/ggplot2/cluster/ggalluvial/pheatmap/mclust/ragg | Provide sessionInfo() in Supplementary Methods. |

**Supplementary Table S1. Cluster-selection, stability, and reproducibility metrics for the two-stage phenotyping workflow.**
Cluster-selection, stability, and reproducibility metrics for the two-stage phenotyping workflow are presented, including silhouette-based results, principal component variance summaries, proportion of ambiguous clustering values, sample-level stability summaries, and adjusted Rand index results.
