# Supplementary material for: Early postoperative voice-change phenotypes after thyroid surgery: a prospective cohort study
Source: Front Endocrinol (Lausanne). 2026 Jun 15;17:1845546. doi: 10.3389/fendo.2026.1845546 (PMC13310725; doi:10.3389/fendo.2026.1845546)
Supplement: Supplementary file 7 [file Table2.docx]

**Supplementary Table S2.**

| **Feature (change from baseline, %)** | ***H* statistic** | **df** | ***P* value** | **ε²** |
| --- | --- | --- | --- | --- |
| Root-mean-square energy change (%) | 67.76 | 2 | < .001 | 0.272 |
| Zero-crossing rate change (%) | 148.39 | 2 | < .001 | 0.605 |
| Spectral centroid change (%) | 176.51 | 2 | < .001 | 0.721 |
| Spectral bandwidth change (%) | 102.08 | 2 | < .001 | 0.414 |
| Mel-frequency cepstral coefficient change (%) | 73.74 | 2 | < .001 | 0.296 |
| Fundamental frequency change (%) | 27.50 | 2 | < .001 | 0.105 |
| Jitter change (%) | 20.87 | 2 | < .001 | 0.078 |
| Shimmer change (%) | 40.11 | 2 | < .001 | 0.157 |
| Harmonics-to-noise ratio change (%) | 20.94 | 2 | < .001 | 0.078 |
| Maximum phonation time change (%) | 3.58 | 2 | .167 | 0.007 |

Abbreviations: df, degrees of freedom; ε², epsilon-squared effect size for the Kruskal–Wallis test. P values are two-sided.

**Supplementary Table S2. POD2 conventional acoustic feature changes by phenotype.**
POD2 changes in conventional acoustic features are presented by phenotype. Continuous variables are reported as median [interquartile range]. Overall comparisons were performed using the Kruskal-Wallis test, with epsilon-squared reported as the effect size. Pairwise comparisons used Wilcoxon rank-sum tests with Benjamini-Hochberg adjustment.
