# Supplementary material for: Early postoperative voice-change phenotypes after thyroid surgery: a prospective cohort study
Source: Front Endocrinol (Lausanne). 2026 Jun 15;17:1845546. doi: 10.3389/fendo.2026.1845546 (PMC13310725; doi:10.3389/fendo.2026.1845546)
Supplement: Supplementary file 8 [file Table3.docx]

Supplementary Table S3. Sensitivity comparison of clustering strategies.

| **Clustering strategy** | **N** | **Final clusters** | **Cluster sizes** | **Final-label mean silhouette** | **PAC 0.1–0.9** | **PAC 0.2–0.8** | **ARI, median [Q1–Q3]** | **Interpretation** |
| --- | --- | --- | --- | --- | --- | --- | --- | --- |
| Primary two-stage workflow | 245 | 3 | 59 / 56 / 130 | 0.231 | 0.402 | 0.251 | 0.707 [0.630–0.877] | Prespecified primary workflow with lower ambiguity and higher resampling reproducibility |
| One-step k = 3 | 245 | 3 | 93 / 60 / 92 | 0.211 | 0.544 | 0.372 | 0.689 [0.328–0.793] | Alternative flat three-cluster solution with higher ambiguity than the primary workflow |
| One-step k = 4 | 245 | 4 | 73 / 45 / 82 / 45 | 0.22 | 0.5 | 0.353 | 0.436 [0.360–0.528] | Four-cluster solution did not improve reproducibility and was not preferred |

Comparison of the primary two-stage workflow with one-step k = 3 and k = 4 alternatives using the same five standardized POD2 spectral change features. ARI is presented as median [Q1–Q3].

Abbreviations: ARI, adjusted Rand index; PAC, proportion of ambiguous clustering; POD2, postoperative day 2; Q1, first quartile; Q3, third quartile.
