# Supplementary material for: Early postoperative voice-change phenotypes after thyroid surgery: a prospective cohort study
Source: Front Endocrinol (Lausanne). 2026 Jun 15;17:1845546. doi: 10.3389/fendo.2026.1845546 (PMC13310725; doi:10.3389/fendo.2026.1845546)
Supplement: Supplementary file 9 [file Table4.docx]

Supplementary Table S4. Surgical and operative characteristics across phenotypes

| **Variable** | **Level** | **Overall**  **(N = 245)** | **Phenotype A**  **(n = 59)** | **Phenotype B**  **(n = 56)** | **Phenotype C**  **(n = 130)** | **P value** |
| --- | --- | --- | --- | --- | --- | --- |
| Extent of thyroidectomy | Unilateral | 145 (59.2%) | 34 (57.6%) | 35 (62.5%) | 76 (58.5%) | 0.843 |
|  | Bilateral | 100 (40.8%) | 25 (42.4%) | 21 (37.5%) | 54 (41.5%) |  |
| Surgical approach | Open | 221 (90.2%) | 53 (89.8%) | 47 (83.9%) | 121 (93.1%) | 0.156 |
|  | Endoscopic | 24 (9.8%) | 6 (10.2%) | 9 (16.1%) | 9 (6.9%) |  |
| Central neck dissection | No | 22 (9.0%) | 1 (1.7%) | 4 (7.1%) | 17 (13.1%) | 0.035 |
|  | Yes | 223 (91.0%) | 58 (98.3%) | 52 (92.9%) | 113 (86.9%) |  |
| Lateral neck dissection | None | 227 (92.7%) | 53 (89.8%) | 51 (91.1%) | 123 (94.6%) | 0.223 |
|  | Unilateral | 15 (6.1%) | 6 (10.2%) | 3 (5.4%) | 6 (4.6%) |  |
|  | Bilateral | 3 (1.2%) | 0 (0.0%) | 2 (3.6%) | 1 (0.8%) |  |
| Operative time, minutes |  | 90 [70, 110] | 90 [75, 112] | 90 [65, 110] | 85 [70, 108] | 0.591 |

Abbreviations: POD7, postoperative day 7.
Table note: Categorical variables are presented as n (%), and operative time is presented as median [interquartile range]. Between-phenotype comparisons were performed using the Pearson chi-square test for categorical variables and the Kruskal-Wallis test for operative time.
