# Supplementary material for: Early postoperative voice-change phenotypes after thyroid surgery: a prospective cohort study
Source: Front Endocrinol (Lausanne). 2026 Jun 15;17:1845546. doi: 10.3389/fendo.2026.1845546 (PMC13310725; doi:10.3389/fendo.2026.1845546)
Supplement: Supplementary file 10 [file Table5.docx]

Supplementary Table S5. Exploratory associations of demographic, imaging, and operative variables with POD7 clinical outcomes

| **Variable** | **Variable type** | **P value for VHI-30 change** | **Test for VHI-30 change** | **P value for VHI-30 responder** | **Test for VHI-30 responder** | **P value for GRBAS Grade**  **(G) change** | **Test for GRBAS Grade**  **(G) change** |
| --- | --- | --- | --- | --- | --- | --- | --- |
| Age, years | Continuous | 0.063 | Spearman correlation | 0.96 | Wilcoxon rank-sum test | 0.143 | Spearman correlation |
| Sex | Categorical | 0.29 | Wilcoxon rank-sum test | 0.027 | Pearson chi-square test | 0.923 | Wilcoxon rank-sum test |
| BMI, kg/m^2^ | Continuous | 0.649 | Spearman correlation | 0.762 | Wilcoxon rank-sum test | 0.519 | Spearman correlation |
| Hashimoto thyroiditis | Categorical | 0.849 | Wilcoxon rank-sum test | 0.035 | Fisher exact test | 0.544 | Wilcoxon rank-sum test |
| Posteriorly located nodule on ultrasonography | Categorical | 0.951 | Wilcoxon rank-sum test | 0.12 | Pearson chi-square test | 0.678 | Wilcoxon rank-sum test |
| Trachea-adjacent nodule on ultrasonography | Categorical | 0.773 | Wilcoxon rank-sum test | 0.145 | Pearson chi-square test | 0.812 | Wilcoxon rank-sum test |
| Maximum nodule diameter on ultrasonography, cm | Continuous | 0.905 | Spearman correlation | 0.765 | Wilcoxon rank-sum test | 0.436 | Spearman correlation |
| Focality | Categorical | 0.976 | Wilcoxon rank-sum test | 0.985 | Pearson chi-square test | 0.158 | Wilcoxon rank-sum test |
| Extent of thyroidectomy | Categorical | 0.816 | Wilcoxon rank-sum test | 0.858 | Pearson chi-square test | 0.673 | Wilcoxon rank-sum test |
| Surgical approach | Categorical | 0.063 | Wilcoxon rank-sum test | 0.543 | Fisher exact test | 0.369 | Wilcoxon rank-sum test |
| Central neck dissection | Categorical | 0.115 | Wilcoxon rank-sum test | 0.326 | Fisher exact test | 0.967 | Wilcoxon rank-sum test |
| Lateral neck dissection | Categorical | 0.142 | Kruskal-Wallis test | 0.505 | Fisher exact test | 0.075 | Kruskal-Wallis test |
| Operative time, minutes | Continuous | 0.166 | Spearman correlation | 0.639 | Wilcoxon rank-sum test | 0.129 | Spearman correlation |
| Phenotype | Categorical | <.001 | Kruskal-Wallis test | <.001 | Fisher exact test | <.001 | Kruskal-Wallis test |

Abbreviations: BMI, body mass index; GRBAS, Grade, Roughness, Breathiness, Asthenia, Strain; POD7, postoperative day 7; VHI-30, 30-item Voice Handicap Index.
Table note: VHI-30 change was calculated as POD7 minus baseline. VHI-30 responder was defined as an increase in VHI-30 score of at least 13 points from baseline to POD7. GRBAS Grade (G) change was calculated as POD7 minus baseline. These analyses were exploratory. Continuous predictors were assessed using Spearman correlation for continuous outcomes and Wilcoxon rank-sum tests for the binary responder outcome. Categorical predictors were assessed using Wilcoxon rank-sum tests or Kruskal-Wallis tests for continuous outcomes and Pearson chi-square or Fisher exact tests for the binary responder outcome, as appropriate.
